# Promoter DNA Methylation Pattern Identifies Prognostic Subgroups in Childhood T-Cell Acute Lymphoblastic Leukemia

**DOI:** 10.1371/journal.pone.0065373

**Published:** 2013-06-06

**Authors:** Magnus Borssén, Lars Palmqvist, Kristina Karrman, Jonas Abrahamsson, Mikael Behrendtz, Jesper Heldrup, Erik Forestier, Göran Roos, Sofie Degerman

**Affiliations:** 1 Department of Medical Biosciences, Pathology, Umeå University, Umeå, Sweden; 2 Department of Clinical Chemistry and Transfusion Medicine, Sahlgrenska University Hospital, University of Gothenburg, Gothenburg, Sweden; 3 Department of Clinical Genetics, University and Regional Laboratories, Skåne University Hospital, Lund University, Lund, Sweden; 4 Institute of Clinical Sciences, Department of Pediatrics, Sahlgrenska University Hospital, Gothenburg, Sweden; 5 Department of Pediatrics, Linköping University Hospital, Linköping, Sweden; 6 Department of Pediatrics, Skåne University Hospital, Lund, Sweden; Dartmouth Medical School, United States of America

## Abstract

**Background:**

Treatment of pediatric T-cell acute lymphoblastic leukemia (T-ALL) has improved, but there is a considerable fraction of patients experiencing a poor outcome. There is a need for better prognostic markers and aberrant DNA methylation is a candidate in other malignancies, but its potential prognostic significance in T-ALL is hitherto undecided.

**Design and Methods:**

Genome wide promoter DNA methylation analysis was performed in pediatric T-ALL samples (n = 43) using arrays covering >27000 CpG sites. Clinical outcome was evaluated in relation to methylation status and compared with a contemporary T-ALL group not tested for methylation (n = 32).

**Results:**

Based on CpG island methylator phenotype (CIMP), T-ALL samples were subgrouped as CIMP+ (high methylation) and CIMP− (low methylation). CIMP− T-ALL patients had significantly worse overall and event free survival (p = 0.02 and p = 0.001, respectively) compared to CIMP+ cases. CIMP status was an independent factor for survival in multivariate analysis including age, gender and white blood cell count. Analysis of differently methylated genes in the CIMP subgroups showed an overrepresentation of transcription factors, ligands and polycomb target genes.

**Conclusions:**

We identified global promoter methylation profiling as being of relevance for subgrouping and prognostication of pediatric T-ALL.

## Introduction

Acute lymphoblastic leukemia (ALL) is the most common malignancy among children. A majority of the ALL cases originates from the B-cell compartment and is characterized by structural genetic aberrations. However, about 15% derive from the T-cell lineage (T-ALL). Over the past 30 years a remarkable improvement in ALL treatment has occurred, but the therapy success rate for T-ALL is lower than for precursor B-cell lymphoblastic leukemia. T-ALL harbors a multitude of genetic alterations, frequently associated with rearrangements of T cell antigen receptor genes with involved fusion genes, such as *TLX1*, *LMO1, LMO2, TAL1* and *LYL1* [1], [2]. Mutations of *NOTCH1, FBXW7*, *PTEN* and *WT1* have also been reported in several studies [1], [3]. These aberrations have provided new insights into the malignant transformation of T-cells, but the prognostic information given by the individual genetic abnormalities is unclear or controversial [4]. Due to the lack of good prognostic markers all T-ALL patients have been enrolled in high intensive treatment programs with substantial therapy related side effects.

Apart from classical genetic aberrations, epigenetics has emerged as an important regulator of cell functions that has been implicated in cancer development [5]. The term “epigenetics” refers to control of gene expression without affecting the actual genetic sequence due to mechanisms like DNA methylation and post translational histone modifications. DNA methylation occurs on CpG (cytosine preceding a guanine) nucleotides distributed across the entire genome but are enriched in CpG islands (CGI). Transformation from a benign to a neoplastic cell is generally associated with a decrease in global CpG site methylation concomitant with an increase in promoter associated CGI methylation. Global demethylation is associated with chromosomal instability and gain of methylation at promoters might lead to silencing of tumor suppressor genes [6].

Further subclassification of hematological malignancies, besides morphologic, immunophenotypic, and genetic features, is of increasing importance in parallel with the development of more target directed therapies. In this context DNA methylation is of potential interest and has appeared as a candidate for subtype discrimination in breast cancer, glioma and colon cancer [7]–[9]. In many studies, methylation status of a limited number of promoter associated CpG sites or promoter regions have been used to define tumors as CGI methylator phenotype positive (CIMP+) or negative (CIMP−). In myelodysplastic syndrome (MDS) CIMP+ patients had increased risk of malignant transformation, shorter survival and could benefit from DNA methyltransferase inhibitor therapy [10], [11]. In ALL, including T-ALL, the methylation pattern of certain CpG site positions in selected promoters were associated with treatment outcome, indicating that methylation on some of these CpG sites was associated with a worse outcome [12]. A Nordic study identified a set of CpG sites discriminating between different forms of ALL based on their methylation status and an association to prognosis was shown in subgroups of B-ALL, but not for T-ALL [13]. Noteworthy, Deneberg and coworkers recently demonstrated, that cytogenetically normal AML cases with a globally methylated promoter profile had a significantly better survival compared with less methylated cases [14]. Our knowledge about global promoter DNA methylation profiles in T-ALL is sparse and its possible role in prognostication and/or subgrouping has not been established.

In the present study we characterized the DNA methylation pattern in diagnostic pediatric T-ALL samples using genome-wide promoter focused methylation arrays identifying two subgroups with highly significant differences in clinical outcome.

## Materials and Methods

### Patient and Control Samples

Between January 1, 1992 and June 30, 2008, 75 infants, children, and adolescents <18 years were diagnosed with T-ALL at the Swedish regional pediatric oncologic centers in Lund (N = 18), Göteborg (N = 34), Linköping (N = 8) and Umeå (N = 15). 46 patients were treated according to the NOPHO (Nordic Society of Pediatric Hematology and Oncology) ALL 1992 protocol and 29 patients according to the NOPHO ALL 2000 protocol [15], [16]. Diagnostic bone marrow material was available for DNA analysis from 43 of the 75 patients (57%) and was included in the promoter methylation investigation. The Regional Ethics Committee in Umeå, Lund and at the Karolinska Institute in Sweden approved the study, and the patients and/or their guardians provided informed consent and during the later years written informed consent. All samples were anonymized in figures and tables. The study was conducted in accordance with the Declaration of Helsinki. The T-ALL diagnosis was in each case based on morphology and flow cytometric immunophenotyping. Cytogenetic aberrations were established by G-band karyotyping and over time FISH analysis was increasingly used.

DNA from age-matched mononuclear normal bone marrow (n = 3) cells, and mitogen (WGA) stimulated primary lymphoblast T-cell cultures (L2 at 5 population doublings (PD) and P7/R2- at 14 PD) derived from mononuclear cells of healthy adult donors were included as controls [17], [18].

### Genome Wide Promoter CpG Methylation Profiling

Genomic DNA from diagnostic T-ALL samples, normal bone marrow and T-cell cultures were isolated by standard procedures and DNA purity and concentration were determined by spectrophotometry (NanoDrop, Thermo Scientific, Wilmington DE, USA). The DNA (500 ng) was bisulfite treated and purified according to manufacturer’s protocol (Zymo EZ DNA methylation kit, Zymo Research, CA, USA). M.S*ssI*-modified DNA (all cytosines methylated) was included as a methylated reference and standard curve in the MethyLight assays. The bisulfite conversion was confirmed using the MethyLight technology for amplification and detection of methylated DNA regions in a iQ™5 Real-Time PCR Detection System (BioRad laboratories, Hercules, CA, USA). A C-LESS-C1 reaction was performed as described by Weisenberger *et al*. 2008 and with the specified primers and probes [19]. Since the C-LESS reaction amplifies both template strands of unconverted genomic DNA but only one strand of bisulfite-converted DNA, the bisulfite-converted DNA will amplify one PCR cycle later than unconverted DNA. In parallel, the *ALU* gene was analyzed with the methylation independent ALU-C4 primer/probe sets [20]. The ALU and C-LESS-C1 PCR reactions were run at 95°C for 10 minutes, followed by 50 cycles of 95°C 15 s, 60°C 60 s. All samples passed these preliminary control analyses before they were applied on the arrays.

Genome-wide promoter CpG site methylation profiling was performed using the Illumina Infinium HumanMeth27K BeadArray (Illumina Inc., San Diego, USA). These arrays generate data for 27578 CpG nucleotides, corresponding to 14473 individual gene promoter regions spanning from −1,000 to +500 from the transcription start site [21]. CpG nucleotides are preferentially located within CpG islands and the majority of CpG sites (∼70%) on the array were classified as being within a CpG island as defined by Takai and Jones relaxed criteria [22]. The methylation assay was performed according to Illumina instructions. Briefly, bisulfite converted DNA was fragmented and amplified with supplied reagents and the pre-processed DNA was hybridized to the arrays. Each CpG site on the array was represented by two site-specific probes, one designed for the methylated locus (M) and another for the unmethylated locus (U). Single-base extension of the probes incorporates labeled ddNTP, which subsequently is stained with a fluorescence reagent. The arrays were scanned using a BeadArray Reader (Illumina). Image processing and intensity data extraction were performed according to Illuminás instructions using the GenomeStudio software (V2010.3) with the methylation analysis module (version 1.8.5). The methylation level for the interrogated locus was determined by calculating the ratio (ß value) of the fluorescent signals from the methylated (M) vs unmethylated (U) sites. ß = Max(M,0)/(Max(M,0)+Max(U,0)+100) ranging in theory from 0, corresponding to completely unmethylated, to 1, representing fully methylated DNA.

**Figure 1:**
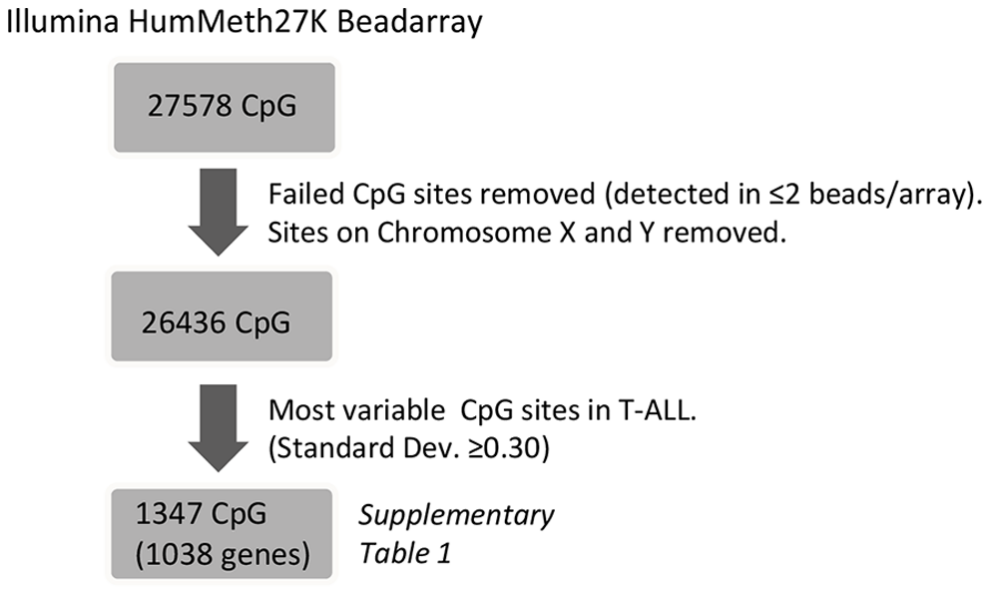
Schematic overview of the methylation array analysis.

As recommended by Illumina technical support, data were not normalized due to the nature of the ß-value calculated as a ratio of the methylated and the unmethylated signal. Empirical testing of background normalization had minute effects on the results. CpG sites that failed (detected in ≤2 beads/array) in one or several arrays were omitted from the analysis. Furthermore, to avoid gender bias, all CpGs located on the X or Y chromosomes were excluded. Analysis was subsequently restricted to the remaining 26436 CpG sites. Unsupervised and supervised hierarchical clustering analysis with the Euclidean distance algorithm was used to create heat maps and dendograms. In order to identify the most variable methylated probes within the T-ALL group a standard deviation ≥0.30 of the β-value across the samples was used as cut-off. A summary over the CpG selection steps is shown in Figure 1. Methylation array data has been deposited to the NCBI Gene Expression Omnibus (GEO) database (GSE42079).

### Verification of Array Data

A subset of 10 T-ALL samples was also separately analyzed at the SNP&SEQ Technology Platform laboratory in Uppsala, Sweden, using a high density array (485577 CpG sites) (HumanMeth450K bead array, Illumina Inc., San Diego, USA), including >90% of the CpG sites on the HumanMeth27K array. A methylated control sample (Human HCT116 DKO methylated DNA, Zymo Research) enzymatically methylated on all cytosines by M.SssI methyltransferase, and a non-methylated (<5%) control double knocked-out for DNA methyltransferases (DNMT1−/− and DNMT3B−/−) (Human HCT116 DKO non-methylated DNA, Zymo Research), were included. A replicate sample was also included to assess inter-assay reproducibility (R^2^ = 0.989). For each sample, 500 ng DNA was bisulfite converted with the EZ-96 DNA Methylation-Gold™ Kit (Zymo Research) according to the instruction manual. 200 ng of bisulfite-converted DNA was applied to each array according to the Illumina HumanMeth450K protocol, arrays were scanned and data analyzed with the GenomeStudio Software.

In addition, nine T-ALL samples were sent to Genome Centre Queen Mary, University of London for targeted pyrosequencing on a selection of gene promoter regions overlapping with specific CpGs in the methylation array, including TAL1 (cg19797376), KLF4 (cg07309102) and HOXD8 (cg15520279). In brief, pyrosequencing was performed according to manufacturers protocols by bisulfite treatment of DNA (EZ DNA Methylation, Zymo Research), followed by PCR amplification and pyrosequencing using PyroMark Gold Q96 Reagents (Qiagen) in the PSQ 96MA instrument with PSQ 96MA software V2.1 (Qiagen). The following set of primers were used: TAL1_F:ATGGGGGTTAGAGAGAGAATGA; TAL1_R:ACCTCCTCAACCAAATCTC; TAL1_seq:GGGGGATTTTAAGGT; HOXD8_F:AGTGATAGTAGTAGTAAGTGGGATTGAT; HOXD8_R:AACAACCCCCCCACAAACCCC; HOXD8_seq:GTTTTGTATTTGGAGTATAG; KLF4_F:AGGTTGTAGAGAAGGAAGTTATAAGTAAG; KLF4_R:CAACAACCTCCCCCACCACTAT; KLF4_ seq:ATACCCCCAAATAAAACTAACTAC.

### Gene Expression Array Analysis and q-RT-PCR Verification

Total RNA was isolated from 17 frozen bone marrow samples using TRIZOL Reagent (Invitrogen, Stockholm, Sweden), according to the manufacturer’s protocol. Concentrations of total RNA were determined by spectrophotometry (NanoDrop, Thermo Scientific, Wilmington, DE, USA) and RNA quality was analyzed (Agilent 2100 Bioanalyzer, Agilent Technologies, Santa Clara, CA, USA). 500 ng of total RNA from each sample was used for cRNA production by the Illumina TotalPrep RNA amplification kit (Ambion Inc, St.Austin, TX, USA) according to the protocol provided. The biotinylated cRNA was quantified (NanoDrop), and the quality of cRNA was evaluated using the RNA 6000 pico kit (Agilent 2100 Bioanalyzer, Agilent Technologies).

A total of 750 ng biotinylated cRNA was used for hybridization to a human HT12 Illumina Beadchip gene expression array (Illumina, San Diego, CA, USA) according to the manufacturer’s protocol. The arrays were scanned using the Illumina BeadArray Reader (Illumina). The Illumina GenomeStudio software was used for data analysis and normalization by the quantile algorithm. Genes with signals below background level were excluded from the analysis, and differentially expressed genes (2-fold) between CIMP subgroups were identified by fold change calculations. Gene expression data was merged with methylation data in the GenomeStudio software and exported to Excel 2010 (Microsoft Office) for visualization. Gene expression array data has been deposited to the NCBI GEO database (GSE41621).

*TAL1* gene expression was verified by q-RT-PCR analysis. Briefly, cDNA was prepared (Superscript III, Invitrogen, Sweden) and PCR performed using TaqMan (Applied Biosystems, Inc., Foster city, CA, USA) gene expression assays for TAL1 (Hs01097987_m1) and TBP (Hs00427620_m1) genes with the TaqMan universal mastermix II, according manufacturers protocol in the ABI Prism 7900HT instrument (Applied Biosystems). A cell line, CCRF-CEM (CCL-119, ATCC; Manassas, VA, USA) was used in 5-fold dilutions as standard curve and the relative TAL1 mRNA level was normalized to the housekeeping gene TBP.

### Statistical and Bioinformatics Analysis

The Statistical Package for the Social Sciences (SPSS Inc., Chicago, IL) software for Macintosh 11 was used for the statistical analyses. Estimates of event free survival (pEFS) and overall survival (pOS) are given at 5 years and were calculated using the Kaplan-Meier method and the different subgroups listed in Table 1 were compared using the log rank test. In Table 1, the chi-square/Fisher’s Exact test was used to compare differences between variables among subgroups; the Mann-Whitney U test was used for continuous variables. For multivariate analysis the Cox proportional-hazard regression model was used, significance evaluated with Log rank. Factors included in the equation were CIMP status, gender, age at diagnosis, and WBC (Table 2). The significance limit for two-sided p-values was set to <0.05 in all tests. Time in first remission (CR1) was defined as time from diagnosis until first event, comprising induction failure, relapse, death of disease, death in remission, or second malignant neoplasm. In the OS analysis, death of any cause was the endpoint. The NOPHO leukemia registry is updated annually and follow-up data were extracted from the registry as of February, 2011.

**Table 1:** Clinical characteristics and outcome in 75 T-ALL of which 43 cases were CIMP classified.

	CIMP+, N = 21	CIMP−, N = 22	Not analysed, N = 32	p value	Total, N = 75
Gender M/F	17/4	19/3	28/4	ns	64/11
Median age (range, years)	8.7 (2.4–16.6)	8.4 (1.6–16.8)	8.8 (2.4–16.8)	ns	8.7 (1.6–16.8)
WBC×10^9^/l (range)	73.0 (0.8–540)	141 (2.0–572)	51.8 (1.1–768)	ns	73.0 (0.8–768)
Relapse (months to relapse)	2 (10–30)	14 (3–91)	6 (3–28)		22 (3–91)
DCR1	1	0	5		6
Ind death	0	1	2		3
RD	0	0	1		1
Death	3	12	12		27
pEFS 5y (SD)	0.86 (0.08)	0.36 (0.10)	0.56 (0.09)	0.001	0.59 (0.06)
pOS 5y (SD)	0.86 (0.08)	0.45 (0.11)	0.62 (0.09)	0.02	0.63 (0.06)

**Table 2:** Multivariate analysis.

Factors in equation	EFS p value (HR)	OS p value (HR)
Gender	ns	ns
Age	ns	ns
WBC	ns	ns
CIMP status	0.006 (1.9)	0.044 (1.64)

The bioinformatics analysis was performed with the GeneGO MetaCore™ software (version 6.7) (Thomson Reuters, New York, USA) and the Database for Annotation, Visualization and Integrated Discovery (DAVID v6.7, http://david.abcc.ncifcrf.gov/) [23]. The MetaCore software was used for “Enrichment by protein function” analysis, cell signaling pathway and network analysis, and the MapEditor tool was used for visualization.

The polycomb target gene lists from Bracken et al. [24] and Lee et al. [25] were compared with the differently methylated gene (DMG) lists in our T-ALL samples using Bioconductor (R statistical programming language). We hypothesized that the proportion of polycomb target genes in our DMG list was as many as expected and tested our hypothesis with a binominal test. Pearson’s Chi-squared test was used to compare the distribution of the number of CpG sites in polycomb target genes on the HumMeth27K array in relation to the entire HumMeth27K array, and to compare the distribution of CpG sites located in polycomb and non-polycomb target genes on the array in relation to the differently methylated CpG sites in T-ALL.

## Results

### Analysis of Promoter DNA Methylation in Pediatric T-ALL Samples and Controls

Genome-wide promoter methylation status was determined by the Illumina HumanMeth27K array in 43 diagnostic pediatric T-ALL samples and 5 controls (normal bone marrow and primary T-cell cultures). The array covers 27578 CpG sites located in 14473 gene promoters, distributed on all chromosomes. CpG sites lacking an average beta value due to ≤2 reported beads/array, as well as CpGs on the X and Y chromosomes were omitted from the analysis to avoid gender-related methylation biases, and analysis was subsequently restricted to the remaining 26436 CpG sites (Figure 1).

The robustness of the results was confirmed by reanalyzing four diagnostic samples with the HumanMeth27K array giving R^2^-values ranging from 0.940 to 0.978 (data not shown). In addition, to independently verify the HumanMeth27K results, 10 T-ALL samples were analyzed with the HumanMeth450K methylation array showing that CpG sites overlapping between the arrays (25978 CpGs) correlated at R^2^ values 0.932+/−0.022. Unsupervised Euclidean clustering of the overlapping CpG sites showed identical clustering for samples analyzed on the HumanMeth27K and 450 K arrays, further substantiating the data obtained (Figure S1).

Furthermore, selected gene promoter regions for TAL1(cg19797376), KLF4 (cg07309102), and HOXD8 (cg15520279), were separately analyzed by pyrosequencing in nine T-ALL samples. The CpG sites analyzed on the HumMeth27K and HumMeth450K arrays were compared with pyrosequencing data on the same position (Figure S5 D, S6 A-B), showing a strong correlation between the methods.

The mean methylation level for the 26436 CpG sites was calculated for each sample. T-ALL samples gave a mean β value of 0.348 (range: 0.284–0.437, n = 43) to be compared with 0.272 (range 0.259–0.294, n = 5) for controls (data not shown). These results indicated a higher global promoter methylation of the T-ALL samples, but also a wide range in methylation levels within the T-ALL group. We focused our analysis on these differences within T-ALL in order to investigate if promoter DNA methylation might be a relevant factor in subgrouping T-ALL.

### Differently Methylated Genes (DMGs) within the T-ALL Group

**Figure 2:**
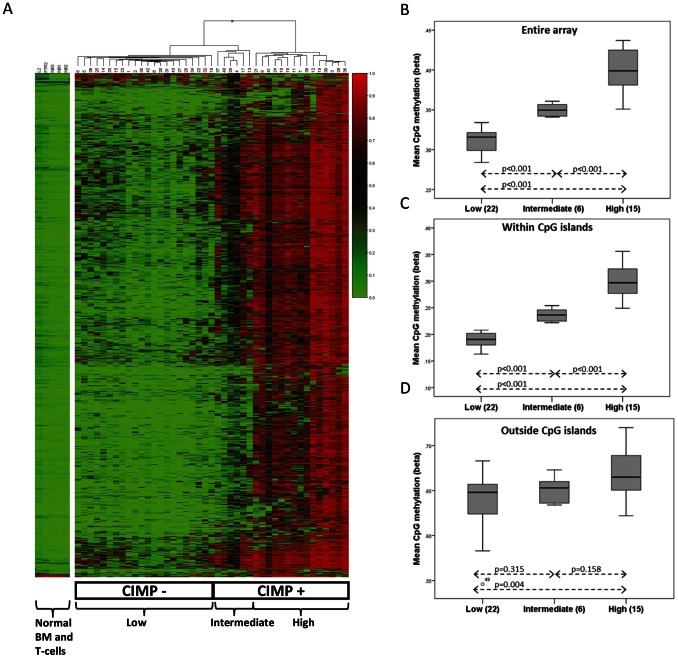
Hierarchical clustering of DNA methylation profiles. A) Unsupervised Euclidean hierarchical clustering was performed using beta values for the 1347 most variable CpG sites in T-ALL (standard deviation ≥0.30). In the dendogram each individual is represented as a vertical row and the specific CpG sites are shown in the horizontal columns. High methylation levels are shown in red and low levels in green, according to the Beta value scale bar in the figure where 1.0 is fully methylated and 0 is unmethylated. The T-ALL methylation subtypes are marked in the figure as low, intermediate and high, and with the merged classification in CIMP− and CIMP+ cases. The methylation status of the 1347 CpG sites of normal bone marrow samples (N1–N3) and two stimulated primary T-cell lines (P7/R2 and L2) are shown next to the hierarchical cluster. B−D) The mean CpG methylation levels within and outside CpG islands were examined. The distribution of mean methylation levels in low (n = 22), intermediate (n = 6) and high (n = 15) methylation subtypes are shown for: B) the entire array (26436 sites), C) within CpG islands (19211 sites), D) outside CpG islands (7225 sites). The significance (independent samples t-test) for differences in methylation between groups is shown.

CpG sites with the largest variation in methylation level within the T-ALL group (standard deviation ≥0.3, 1347 CpG sites) were selected for further examination (Table S1). Subsequent hierarchical cluster analysis of these 1347 CpGs subdivided the samples into three methylation groups, designated low (n = 22), intermediate (n = 6) and high (n = 15) (Figure 2A). The normal bone marrow samples and stimulated T-cells showed a low methylation profile and were similar to the CIMP− samples (Figure 2A). The distribution of mean CpG methylation levels (26436 CpG sites) differed significantly between the groups (p≤0.001, respectively) (Figure 2B). The deviation was more obvious for CpG sites located within CpG islands (CGI) (n = 19211) compared to CpGs outside CGIs (n = 7225) (Figure 2C–D). However, the methylation level was generally higher in CpGs outside islands. Seventy percent of the CpGs covered in the HumanMeth27K array were located within CGIs, but among the DMGs (1347 sites) a majority (98%) was located within CGIs, indicating a preferential methylation (or demethylation) of these sites.

### Treatment Outcome and Promoter Methylation at Diagnosis

**Figure 3:**
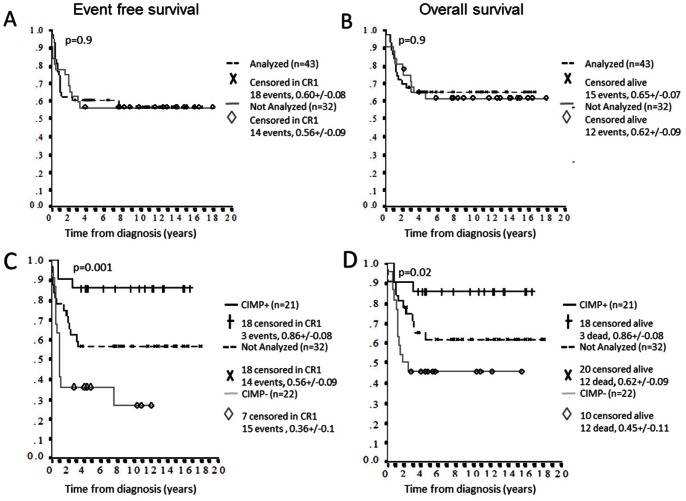
Event free survival (EFS) and overall survival (OS) in methylation subgroups in T-ALL. The 5 year EFS (A) and OS (B) for 43 T-ALL samples analyzed for CpG methylation and 32 T-ALL patients not analyzed but diagnosed during the same time-period showed no differences (p = 0.9 and p = 0.9 respectively). The total patient group (n = 75) had an EFS 5y of 0.59 (SE = 0.06) and OS 5y of 0.63 (SE = 0.06). CIMP+ (n = 21) and CIMP− (n = 22) patients differed significant regarding both 5 year EFS (C) and OS (D) (p = 0.001 and p = 0.02, respectively), with cases lacking methylation data forming an intermediate group.

The T-ALL samples included were diagnosed between years 1992 and 2008 and during this time period two different treatment protocols were used, NOPHO ALL 1992 and 2000. Event free and overall survival did not differ between these two protocols (p = 0.99, and p = 0.3, respectively) (Figure S2 A-B). The entire cohort (n = 75) diagnosed with T-ALL during this time period had an EFS 5y of 0.59 (SE = 0.06) and OS 5y of 0.63 (SE = 0.06) with no difference for patients analyzed for methylation (n = 43) and cases not analyzed (n = 32) (p = 0.9 for both) (Figure 3A–B). Thereby we conclude that our study material was unbiased and had an expected clinical outcome for T-ALL patients during this time period [16].

The T-ALL methylation groups, low (n = 22), intermediate (n = 6) and high (n = 15) (Figure 2A) were examined for clinical outcome. The intermediate and high methylation groups showed no significant difference in outcome regarding EFS and OS (p = 0.25 for both) and were merged into one group denominated CIMP+ (Figure S2 C–D). Accordingly, cases with low methylation were called CIMP− (Figure 2A).

Interestingly, using this subclassification CIMP+ patients had significantly better EFS (86% vs. 36%, p = 0.001) and OS (86% vs. 45%, p = 0.02) compared to CIMP− patients (Figure 3C–D). To confirm that our samples (n = 43) were representative of the entire cohort of patients (n = 75), CIMP+ (n = 21) and CIMP− (n = 22) cases were compared to the 32 T-ALL patients diagnosed during the same time period and for whom DNA and subsequently CIMP data were missing. Clinical data regarding gender, age, WBC, and outcome for the three groups are given in Table 1. No differences in distribution regarding sex, age or white cell count were observed between the CIMP+ and CIMP− groups (Table 1). The median observation time for patients in continuous CR1 was 127 months (range 32–214 months). The differences in EFS and OS between the CIMP +/− groups were retained in multivariate analysis including WBC, age, gender and CIMP status (p = 0.006 and p = 0.044 respectively) (Table 2). Cases lacking methylation data formed an intermediate group between the CIMP+ and CIMP− groups (Figure 3C–D).

### Polycomb Target Genes, Transcription Factors, and Genes in ATP Metabolism were Preferentially Methylated in CIMP+ Samples

To identify potential genes and cellular processes that were connected to the CIMP groups we used the GeneGO metacore software and the DAVID Functional Annotation Tool. The result showed that 1038 genes corresponding to the 1347 differently methylated CpG sites described above were most significantly associated with sequence specific binding, transcription regulator activity, protein binding, and promoter binding (data not shown).

**Figure 4:**
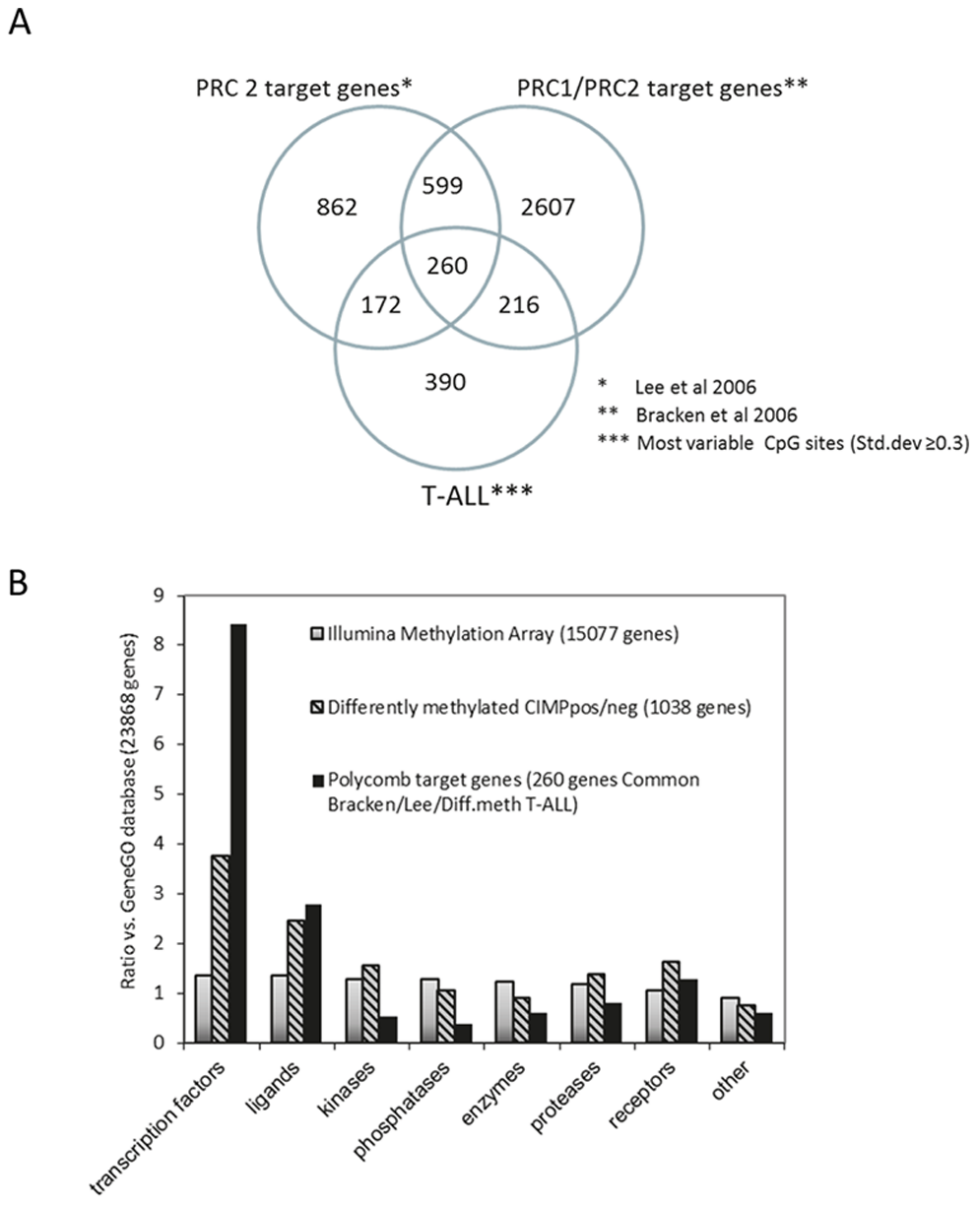
Differently methylated genes in T-ALL are overrepresented by transcription factors and ligands and enriched for polycomb target genes. A) Lists of polycomb target genes identified in human embryonic fibroblasts and embryonic stem cells were compared with the DMGs in T-ALL in a Venn diagram. 260 genes were commonly present in these lists. B) The most variable CpG sites in T-ALL (1347 CpGs/1038 genes) and the 260 common polycomb genes identified in Figure 4A were evaluated for protein function, and compared with the protein function distribution of genes within the GeneGO database (23868 genes) and the Illumina methylation array (15077 genes). Transcription factors and ligands were overrepresented in the DMG in T-ALL compared with the distribution within the database and the entire methylation array.

Our initial GeneGO Metacore analysis of DMGs indicated an overrepresentation of genes associated with the polycomb repressive complexes (PRC) 1 and 2. This is in line with recent reports on AML as well as solid tumor types where hypermethylation of PRC target genes has been observed [14], [26], [27]. To further investigate this we compared previously published lists of PRC target genes identified in human embryonic stem cells (Lee et al.) [25] and human embryonic fibroblasts (Bracken et al.) [24] with our list of most variable gene promoters in T-ALL (n = 1038) (Figure 4A). A high proportion of genes (62%) in our list were identified as polycomb target genes by Lee et al. and/or Bracken et al. The number of PRC target genes in our list was much higher than expected from a random selection (p<0.0001), indicating a preferential methylation of these genes (Figure 4A). We analyzed if the HumMeth27K methylation array was biased regarding the number of CpG sites measured per gene. The Lee and Bracken polycomb target gene lists were combined and compared with the entire HumMeth27K array for distribution of 1, 2 and ≥3 CpG sites per gene. The distribution was comparable, but with a slightly higher number of CpGs in polycomb target genes on the array. However, there was a highly significant overrepresentation of polycomb target genes among the differently methylated CpG sites in T-ALL that cannot be explained by the small array bias (Figure S3 A and S3 B).

Furthermore, the differently methylated genes in T-ALL (1038 genes) and the 260 common PRC target genes in Bracken/Lee/T-ALL (Figure 4A) were evaluated for protein function by enrichment analysis using the Metacore software and compared with genes in the GeneGo database (n = 23868) and in the methylation array (n = 15077). The distribution of genes regarding protein function was similar in the array and in the GeneGO database. However, the DMGs in our T-ALL samples were clearly overrepresented by transcription factors and ligands, 3.8 and 2.5 fold more abundant than expected (Figure 4B). Small or no differences were seen in the following subgroups: kinases, phosphatases, enzymes, proteases and receptors (Figure 4B). The overrepresentation of transcription factors and ligands was further strengthened when focusing on the 260 polycomb target genes (8.4 and 2.8 fold increase, respectively) (Figure 4A–B).

**Figure 5:**
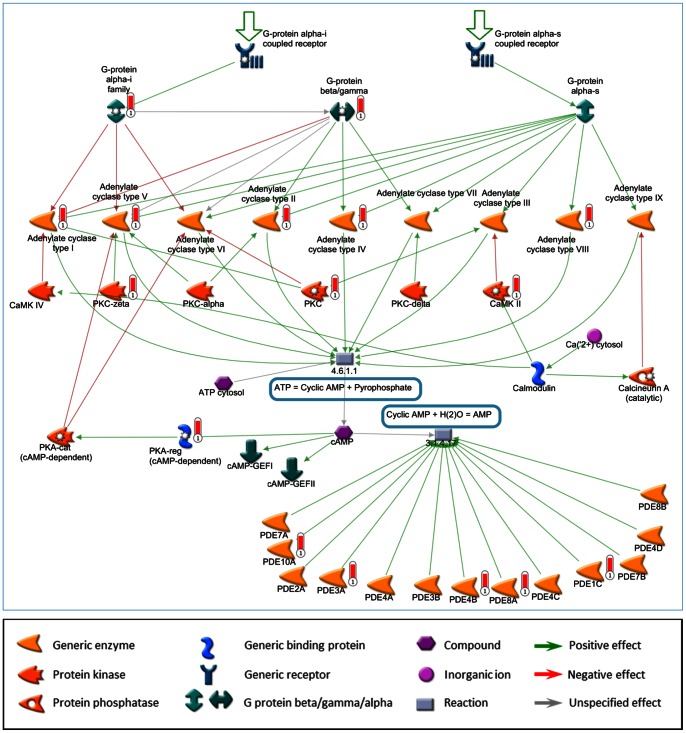
Genes in cAMP signaling and ATP metabolism among the DMGs in T-ALL. A cAMP signaling and ATP metabolism network was created in the MapEditor tool of the Metacore software, and the list of differently methylated genes (DMGs) (1347 CpG/1038 genes, std. dev ≥0.3 within T-ALL) was applied on the network. Each red thermometer highlights a DMG within the network.

Focused analysis of cell processes and metabolic pathways revealed a large number of genes involved in ATP metabolism and cAMP signaling pathways to be differently methylated within the T-ALL group (Figure 5). These genes were more methylated in the CIMP+ group compared to the CIMP− group (data not shown). These genes included adenylate cyclases (*ADCY1, ADCY2, ADCY4, ADCY5* and *ADCY8*) and phosphodiesterases (*PDE10A, PDE3A, PDE4B, PDE8A,* and *PDE1C*), genes that are distributed on different chromosomal locations.

### Promoter Methylation in Relation to Gene Expression

In order to examine the correlation between promoter methylation and gene expression we performed gene expression array analysis (Illumina HT-12) on 17 T-ALL samples. A significant (Spearman correlation Rho −0.260, p<0.001) inverse correlation was observed between promoter methylation and gene expression (Figure S4 A).

By combining the list of differently methylated genes (1038 genes) with a list of differently expressed genes (2-fold up/down, 405 genes), 39 genes including *SOCS2, DCHS1, EPHX2, ATP9A, MARCKS, SIX6, TFAP2C* and *ANXA5* were identified as differently methylated *and* differently expressed (Figure S4 B). The majority of these 39 genes showed a negative correlation between methylation and gene expression, i.e. high promoter methylation was associated with low expression. Since only 47% of the DMGs where expressed above background signal level in the gene expression array, evaluation of a possible influence of promoter methylation on gene expression was not feasible for all genes, *i.e*. the majority of the cAMP/ATP signaling pathway genes.

We next focused our analysis on selected genes previously associated with T-ALL development, including *TAL1, LYL1, TLX1* and *TLX3*. None of these genes were identified as DMGs between CIMP subgroups. However, *TAL1* gene expression (HT-12 array) was negatively correlated (R^2^ = 0.611) with *TAL1* methylation levels (TargetID cg19797376, HumMeth27K array) in 17 TALL samples (Figure S5 A). We verified *TAL1* gene expression levels in the array by q-RT-PCR analysis in six samples (R^2^ = 0.972) (Figure S5 B). Detailed analysis of the HumanMeth450K methylation array (including 36 CpG sites distributed at different locations in the TAL1 promoter and gene body) showed that methylation of CpG sites 1–1500 nucleotides upstream of transcription start site (TSS) was associated with gene expression, in contrast to CpG sites located within the TAL1 gene body (Figure S5 C). Methylation level of the TAL1 CpG site (cg19797376) located close to transcription start site (TSS 200 region) was verified by repeated array analysis and pyrosequencing (Figure S5 D).

An early T cell precursor (ETP) gene expression signature associated with a worse prognosis in T-ALL was identified by Gutierrez *et al*. in 2010 [28]. We used this signature for analysis of the 17 T-ALL samples studied in the gene expression array. However, the ETP signature did not discriminate CIMP+ from CIMP− individuals (Figure S7).

## Discussion

In the present study we demonstrated a prognostic significance of global promoter DNA methylation status at diagnosis in T-ALL. We observed a high variability in CpG site promoter methylation between individual T-ALL samples, which could be subdivided as CIMP+ or CIMP− experiencing large differences in clinical outcome. Previous studies indicate that a CIMP phenotype exists in T-ALL, but are inconclusive regarding a potential connection to prognosis [12], [13], [29]–[31]. One explanation for the diverging reports is the lack of a common CIMP definition and that diverse methodologies have been used. We utilized an array approach to study genome-wide promoter methylation [32], [33] whereas other laboratories have studied a selection of few promoter CpGs [34]. Our classification was based on CpG sites (n = 1347) with the most variable methylation levels within the T-ALL group and revealed a significantly better 5 year OS (p = 0.02) for the CIMP+ T-ALL group (86%) compared with the CIMP− group (45%). Since relapse is associated with poor outcome EFS was very similar to OS, 86% in CIMP+ compared with 36% in CIMP− group. Interestingly, an association between global promoter methylation and good prognosis has recently been observed in both childhood pre-B ALL and in cytogenetically normal adult AML using the same array platform as in the present study [14], [35]. The similar findings in two clinically separate diseases strongly suggest that promoter methylation status can give clinically useful information in separate hematological malignancies. It is notable that the CIMP− T-ALL samples experiencing a worse outcome had a similar methylation profile as normal bone marrow cells and stimulated T-cells. However, in order to compare T-ALL samples with normal samples of the same cellular origin, purified pre-T cells are needed.

DMGs distinguishing CIMP− from CIMP+ samples were in our samples enriched for targets of the polycomb complexes (PRC 1 and 2). Theses complexes have chromatin-modifying capacity, influence CpG methylation and are involved in regulation of developmental and differentiation processes [24], [25]. PRC members are commonly deregulated in cancer, including leukemia and lymphoma [36], [37]. Recently whole-genome sequencing of early T-cell precursor ALL identified alterations in PRC2 genes, including SUZ12, EZH2 and EED [38]. In another study, mice with bi-allelic deletions of EZH2 showed high frequency of T-ALL and low levels of histone H3K27 tri-methylation [39].We analyzed DMGs in T-ALL according to their protein function and observed an overrepresentation of transcription factors and ligands compared with an expected random event. The overrepresentation was even stronger when the analysis was restricted to DMGs overlapping with polycomb target genes identified in embryonic stem cells and embryonic fibroblasts [24], [25]. Lee et al showed a preferential binding of SUZ12, the DNA binding component of PRC2, to transcription factors associated with developmental processes [25]. Accordingly, the molecular function of the DMGs in T-ALL was strongly associated with sequence specific binding, transcription regulator activity, protein binding and promoter binding.

One interesting question is if the increased methylation in CIMP+cases is random or not [40]. Our analysis of DMGs indicated a nonrandom process with preferential methylation of transcription factors, ligands and polycomb targets genes for at least specific sets of genes. Furthermore, methylation seemed to be favored at CpG sites located within CpG islands. In accordance, previous studies have shown that CpG sites within CpG islands are more prone to undergo tumor specific methylation compared to CpG sites outside islands [41].

One important issue is the biological background to the association between DNA methylation and clinical outcome, which we only can speculate upon. It is obvious that the CIMP− subgroup with a poor outcome is more resistant to the treatment received compared to CIMP+ ALL. On the other hand, the better outcome for CIMP+ cases might suggest that specific methylation events are important for a therapy sensitive phenotype. Combinations of cytotoxic drugs are used in pediatric T-ALL therapy targeting a wide range of cellular processes in order to induce DNA damage and cell death [16]. The bioinformatics analysis revealed that many DMGs in CIMP+ T-ALL were genes involved in ATP metabolism and cAMP signaling processes, which have been considered as potential targets for cancer therapy [42]–[44]. However, the relevance of these methylation changes for therapy response in T-ALL needs further studies.

In concordance with other studies we observed a significant negative and weak correlation between methylation and gene expression at a global level [14]. The rather weak global correlation has also been previously observed [14], [45], [46]. Jung *et al.* combined methylation data from the Illumina Golden Gate methylation array (1505 CpG sites/807 genes) with Affymetrix gene expression data in 193 multiple myeloma samples and found only 2.1% of the CpG sites to be correlated with gene expression [45]. The correlation between methylation and gene expression might also depend on where the CpG sites are located, e.g. within/outside CpG islands and the CpG site gene location (e.g. promoter, gene body) [47]. The number of CpGs per promoter in the 27K array did not permit a detailed analysis, but future analysis by high resolution methylation arrays or next generation sequencing might reveal a deeper understanding of the relation between methylation alterations and gene expression. However, we identified several genes with a negative correlation between gene expression and promoter CpG methylation, including EPHX2, DSCHS1, MARSCS, ANXA5, and the T-ALL associated TAL1 gene. Whether or not these genes contributed to the poor prognosis in the CIMP− group remains to be evaluated. It has previously been shown that T-ALL cases with an ETP phenotype, representing up to 15% of all T-ALL cases, have a worse prognosis [28], [38], [48]. We analyzed the gene expression profile associated with the ETP signature and found no association to CIMP status.

In conclusion, our study identified two CIMP groups of T-ALL with significantly different prognosis regarding risk of relapse and overall survival. Few prognostic T-ALL markers are available today and the potential for CIMP status as a predictor of clinical outcome and as a guide for therapy seems promising but needs to be further investigated in larger patient cohorts.

## Supporting Information

Figure S1 **Hierarchal clustering of 10 T-ALL samples analyzed with the HumanMeth27K and 450 K arrays.** Ten T-ALL samples were analyzed on both the HumanMeth27K and HumanMeth450K Illumina methylation arrays. Unsupervised Euclidean hierarchical clustering of the 25978 overlapping CpG sites covered by both arrays in the A) HumanMeth27K and B) HumanMeth450K arrays. Methylation groups identified in Figure 2A is shown in the figure.
(TIF)

Figure S2 **Event free survival (EFS) and overall survival (OS) in T-ALL.** EFS (A) and OS (B) for the 2 different treatment protocols used during years 1992–2008 at which the diagnosis T-ALL samples were collected showing no significant difference between these protocols (p = 0.99 and p = 0.3, respectively). EFS (C) and OS (D) for the methylation subgroups (low n = 22, intermediate n = 6, and high n = 15) identified by hierarchal clustering of the most variable CpG sites in T-ALL (1347 CpGs, Figure 2A). The intermediate and high methylation subgroups showed no difference regarding these parameters (EFS p = 0.25, OS p = 0.25) while the low methylation subgroup differed significantly from these groups (EFS low/high p = 0.005, low/intermediate p = 0.01). The intermediate and high methylation groups were hereafter collectively grouped as CIMP+ and the low group CIMP−.
(TIF)

Figure S3 **Distribution of CpG sites on the HumMeth27K array.** A) The HumanMeth27K array was examined for possible bias regarding the number of CpG sites in polycomb target genes compared to the entire array. The Lee et al. (2006) and Bracken et al. (2006) polycomb target gene lists were combined and compared with the entire HumMeth27K array for distribution of 1, 2 and ≥3 CpG sites per gene. B) The distribution of CpG sites located in polycomb and non-polycomb target gene promoters on the HumMeth27K array was compared with the distribution of the differently methylated CpG sites within T-ALL. Pearsońs Chi squared test was used to test the distribution in both analyses.
(TIF)

Figure S4 **Correlation of gene expression with CpG promoter methylation.** A) Mean CpG methylation levels (beta-value, x-axis) is plotted against mean gene expression levels (log average signal, y-axis) from 17 T-ALL samples. Gene expression data below background level was excluded from the analysis and the remaining 18500 CpG sites is shown. Spearman correlation Rho = −0.260, P<0.001. B) Examples of 8 selected genes identified as differently methylated (T-ALL Std. Dev. ≥0.3, 1347 sites) and differently expressed (2-fold up/down CIMP+/−, 405 genes). Gene expression (log Average signal) is shown on y-axis, and CpG methylation (beta-value) on x-axis for 17 T-ALL patients, and R^2^correlation coefficient shown in figure.
(TIF)

Figure S5 **TAL1 promoter methylation and gene expression.** A) TAL1 gene expression (Average signal, HT12 array) and CpG methylation (Average beta, HumanMeth27K array) correlated at R^2^ = 0.611. B) TAL1 gene expression was verified by TaqMan gene expression q-RT-PCR analysis in 6 samples. Array data and q-RT-PCR data correlated at R^2^ = 0.972. C) Detailed analysis of mean CpG site methylation by the HumanMeth450K array in promoter regions; Gene body, TSS200 (0–200 nt upstream of transcription start site) and TSS1500 (200–1500 nt upstream of TSS) and in correlation to the *TAL1/TBP* gene expression ratio. D) Verification of the TAL1 CpG site (cg19797376) by pyrosequencing. Methylation status (% methylated) revealed by pyrosequencing was compared with HumanMeth27K and HumanMeth450K methylation status on the same CpG position.
(TIF)

Figure S6 **Verification of array data by pyrosequencing.** The methylation status of selected gene promoters, HOXD8 (cg15520279), and KLF4 (cg07309102), was determined by pyrosequencing and compared with the HumMeth27K array data.
(TIF)

Figure S7 **ETP gene signature analysis.** The early T cell precursor (ETP) gene expression signature identified by Gutierrez *et al*. in 2010 [28] was used for analysis of 17 T-ALL samples analyzed by the HT-12 gene expression array. The heat map shows log. Average signal of the ETP associated genes for each sample. Euclidean hierarchical cluster analysis did not discriminate CIMP+ from CIMP− samples.
(TIF)

Table S1 **Most variable (Std.dev. ≥0.3) CpGs (n = 1347) in T-ALL (n = 43).**
(XLSX)
