# Supplementary figures and images for: Promoter DNA Methylation Pattern Identifies Prognostic Subgroups in Childhood T-Cell Acute Lymphoblastic Leukemia
Source: PLoS One. 2013 Jun 6;8(6):e65373. doi: 10.1371/journal.pone.0065373 (PMC3675104; doi:10.1371/journal.pone.0065373)

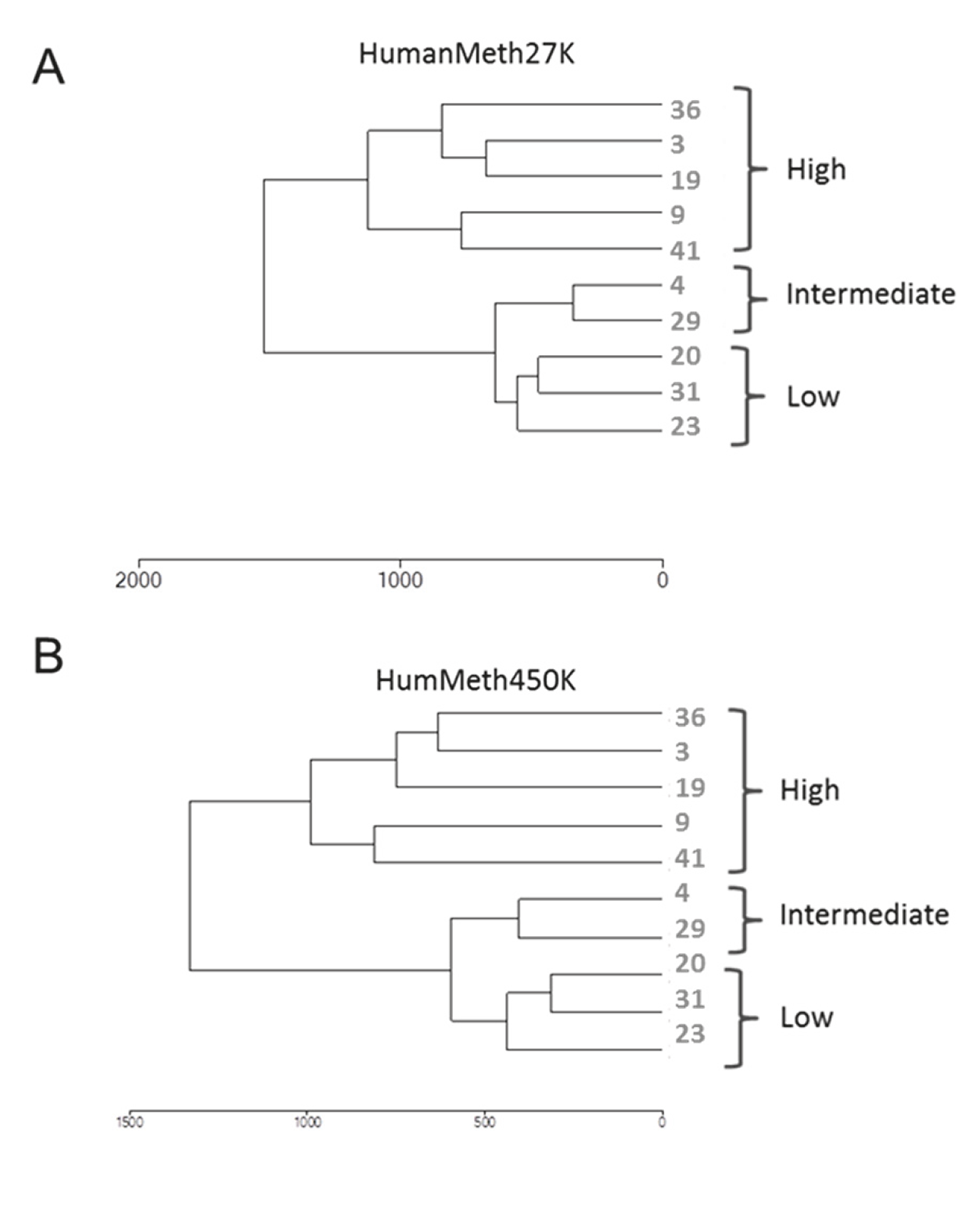

Supplement: Figure S1 — Hierarchal clustering of 10 T-ALL samples analyzed with the HumanMeth27K and 450 K arrays. Ten T-ALL samples were analyzed on both the HumanMeth27K and HumanMeth450K Illumina methylation arrays. Unsupervised Euclidean hierarchical clustering of the 25978 overlapping CpG sites covered by both arrays in the A) HumanMeth27K and B) HumanMeth450K arrays. Methylation groups identified in Figure 2A is shown in the figure. (TIF) [file pone.0065373.s001.tif]

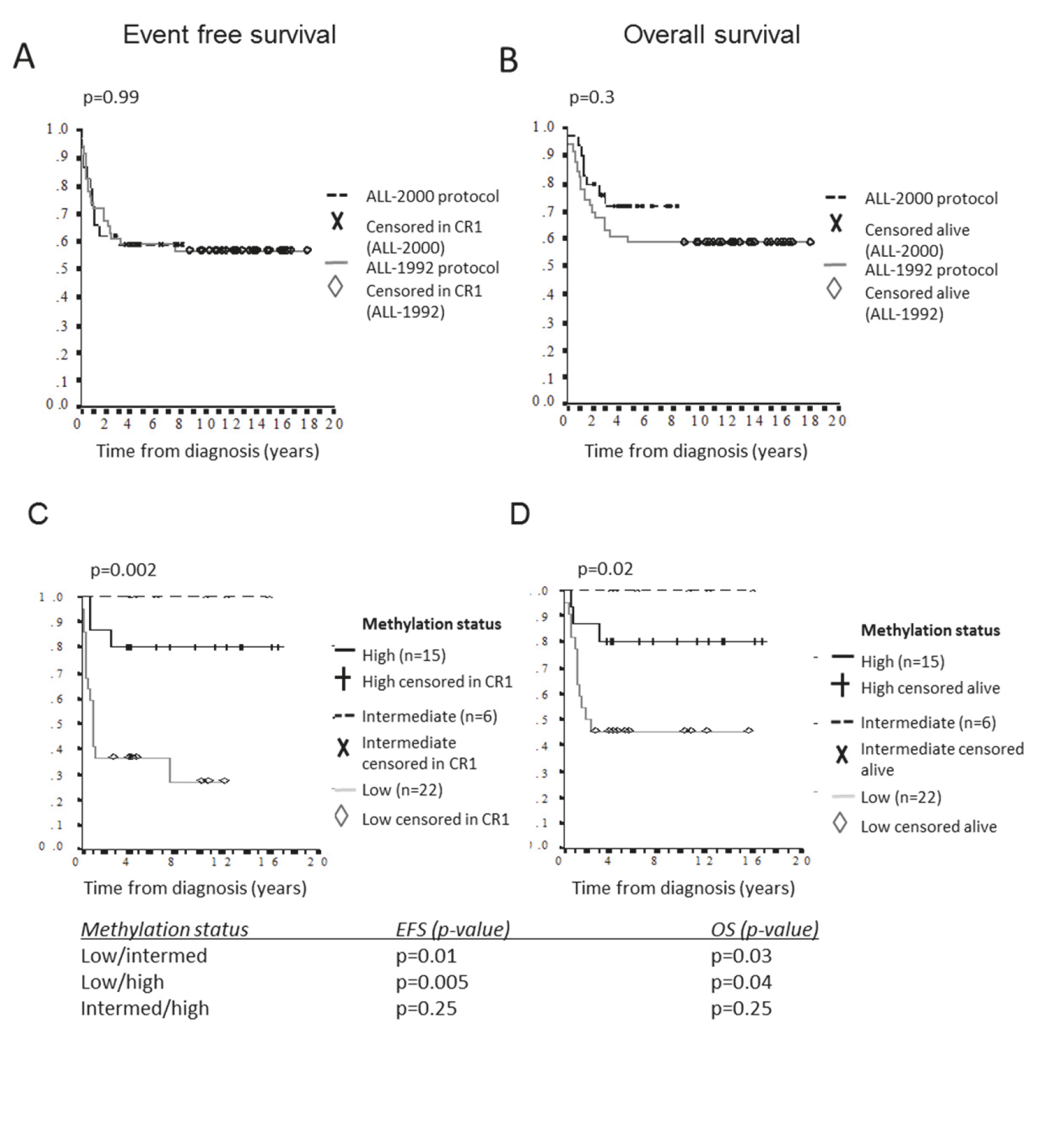

Supplement: Figure S2 — Event free survival (EFS) and overall survival (OS) in T-ALL. EFS (A) and OS (B) for the 2 different treatment protocols used during years 1992–2008 at which the diagnosis T-ALL samples were collected showing no significant difference between these protocols (p = 0.99 and p = 0.3, respectively). EFS (C) and OS (D) for the methylation subgroups (low n = 22, intermediate n = 6, and high n = 15) identified by hierarchal clustering of the most variable CpG sites in T-ALL (1347 CpGs, Figure 2A). The intermediate and high methylation subgroups showed no difference regarding these parameters (EFS p = 0.25, OS p = 0.25) while the low methylation subgroup differed significantly from these groups (EFS low/high p = 0.005, low/intermediate p = 0.01). The intermediate and high methylation groups were hereafter collectively grouped as CIMP+ and the low group CIMP−. (TIF) [file pone.0065373.s002.tif]

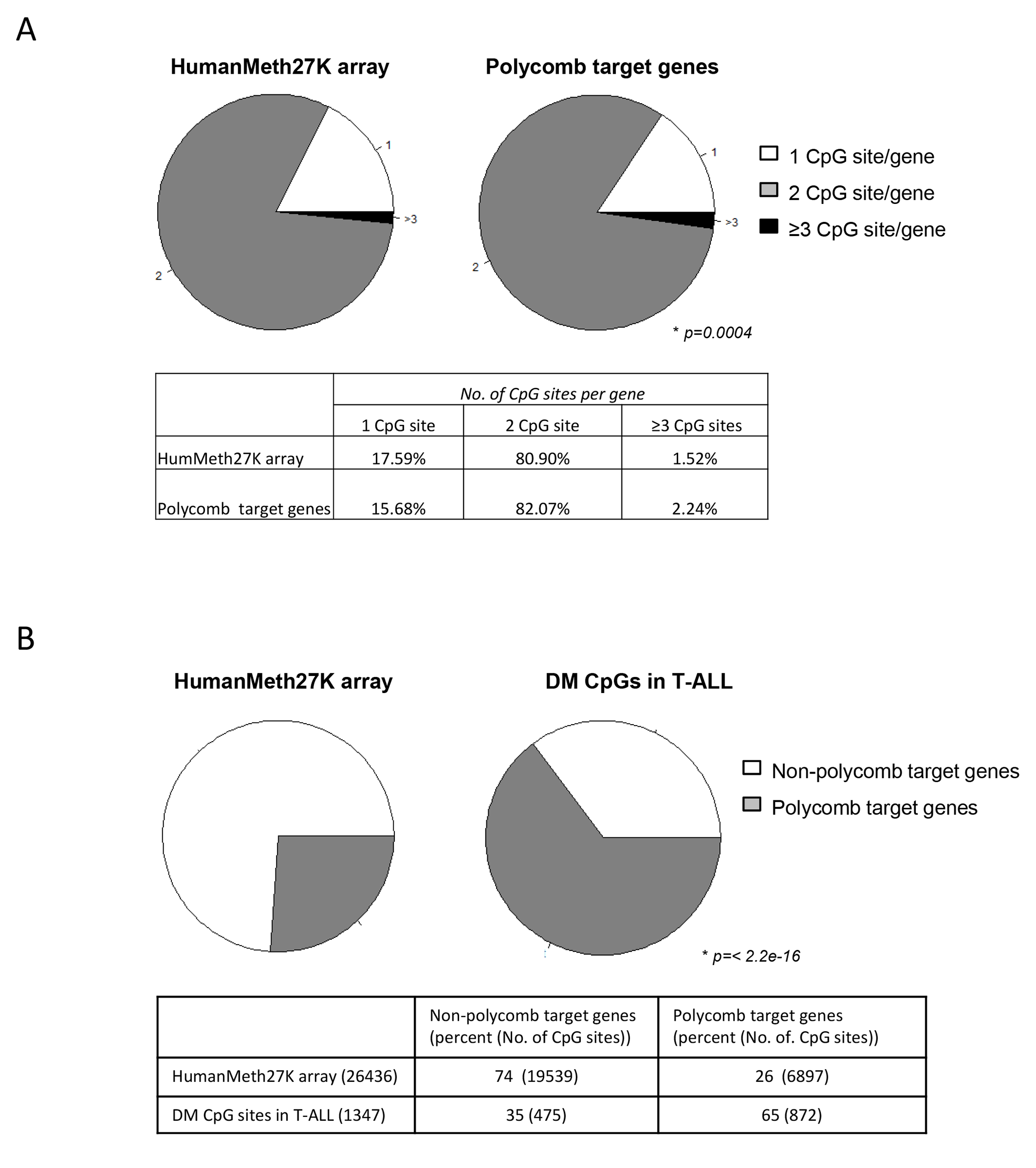

Supplement: Figure S3 — Distribution of CpG sites on the HumMeth27K array. A) The HumanMeth27K array was examined for possible bias regarding the number of CpG sites in polycomb target genes compared to the entire array. The Lee et al. (2006) and Bracken et al. (2006) polycomb target gene lists were combined and compared with the entire HumMeth27K array for distribution of 1, 2 and ≥3 CpG sites per gene. B) The distribution of CpG sites located in polycomb and non-polycomb target gene promoters on the HumMeth27K array was compared with the distribution of the differently methylated CpG sites within T-ALL. Pearsońs Chi squared test was used to test the distribution in both analyses. (TIF) [file pone.0065373.s003.tif]

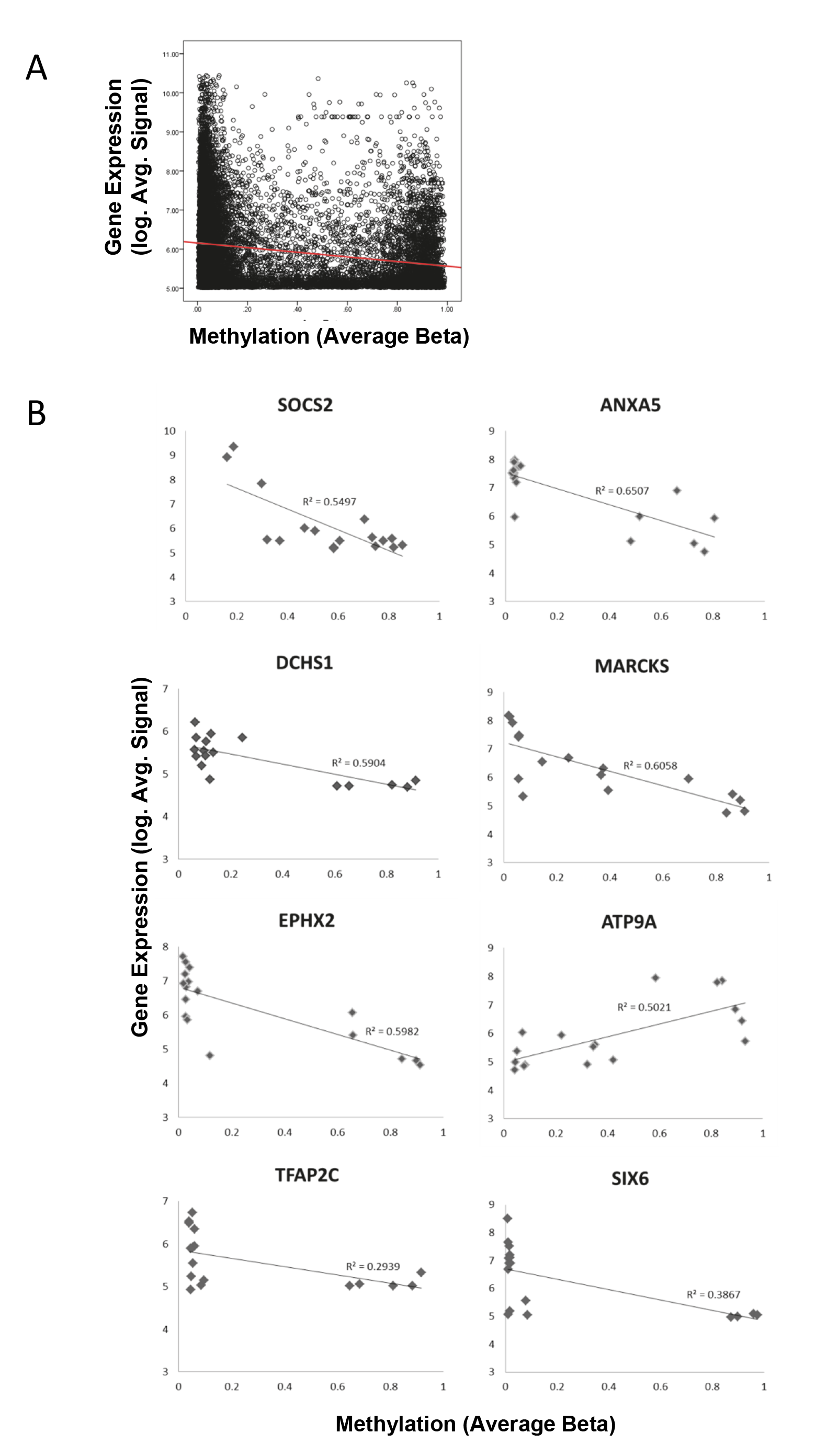

Supplement: Figure S4 — Correlation of gene expression with CpG promoter methylation. A) Mean CpG methylation levels (beta-value, x-axis) is plotted against mean gene expression levels (log average signal, y-axis) from 17 T-ALL samples. Gene expression data below background level was excluded from the analysis and the remaining 18500 CpG sites is shown. Spearman correlation Rho = −0.260, P<0.001. B) Examples of 8 selected genes identified as differently methylated (T-ALL Std. Dev. ≥0.3, 1347 sites) and differently expressed (2-fold up/down CIMP+/−, 405 genes). Gene expression (log Average signal) is shown on y-axis, and CpG methylation (beta-value) on x-axis for 17 T-ALL patients, and R2correlation coefficient shown in figure. (TIF) [file pone.0065373.s004.tif]

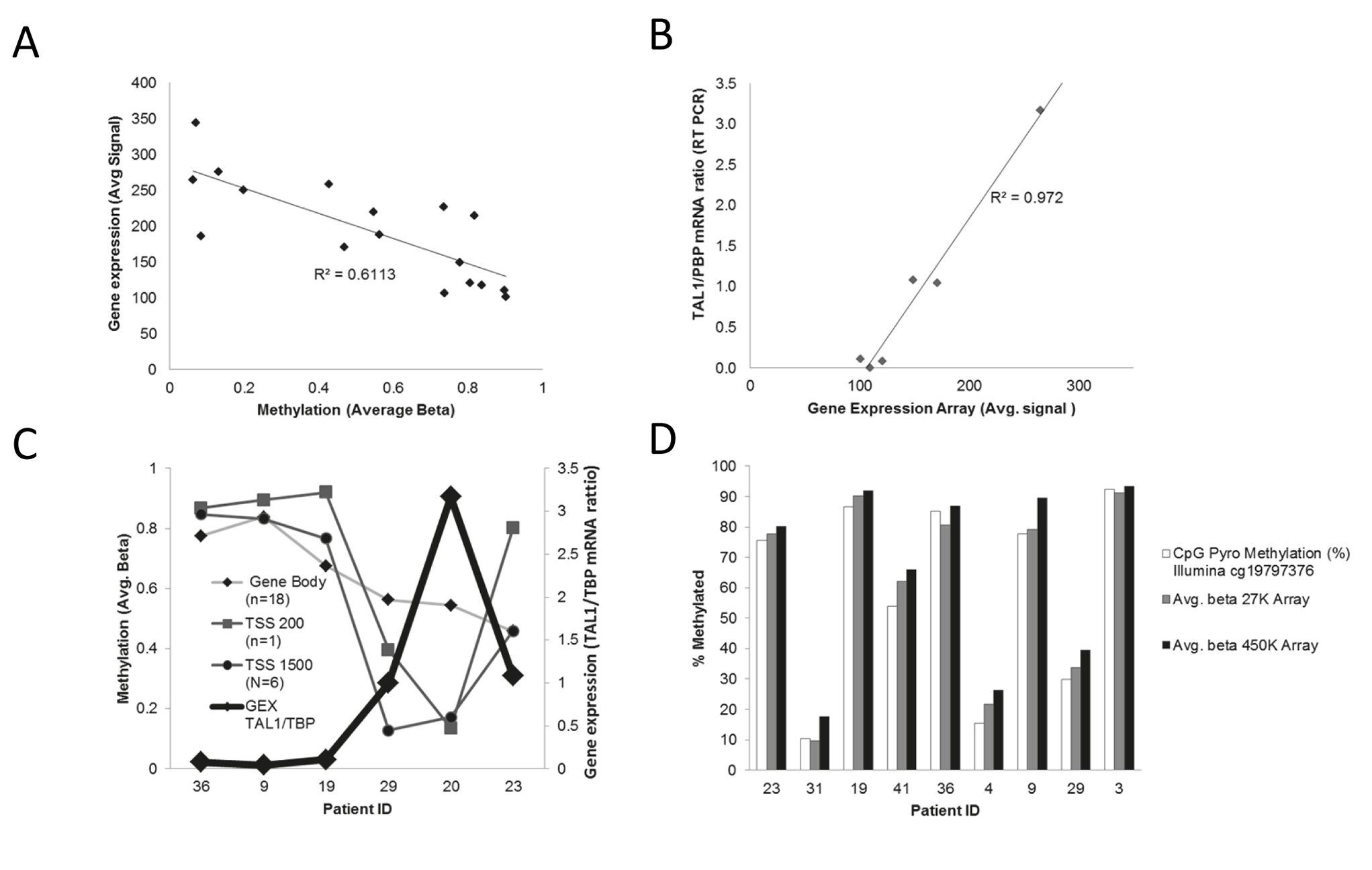

Supplement: Figure S5 — TAL1 promoter methylation and gene expression. A) TAL1 gene expression (Average signal, HT12 array) and CpG methylation (Average beta, HumanMeth27K array) correlated at R2 = 0.611. B) TAL1 gene expression was verified by TaqMan gene expression q-RT-PCR analysis in 6 samples. Array data and q-RT-PCR data correlated at R2 = 0.972. C) Detailed analysis of mean CpG site methylation by the HumanMeth450K array in promoter regions; Gene body, TSS200 (0–200 nt upstream of transcription start site) and TSS1500 (200–1500 nt upstream of TSS) and in correlation to the TAL1/TBP gene expression ratio. D) Verification of the TAL1 CpG site (cg19797376) by pyrosequencing. Methylation status (% methylated) revealed by pyrosequencing was compared with HumanMeth27K and HumanMeth450K methylation status on the same CpG position. (TIF) [file pone.0065373.s005.tif]

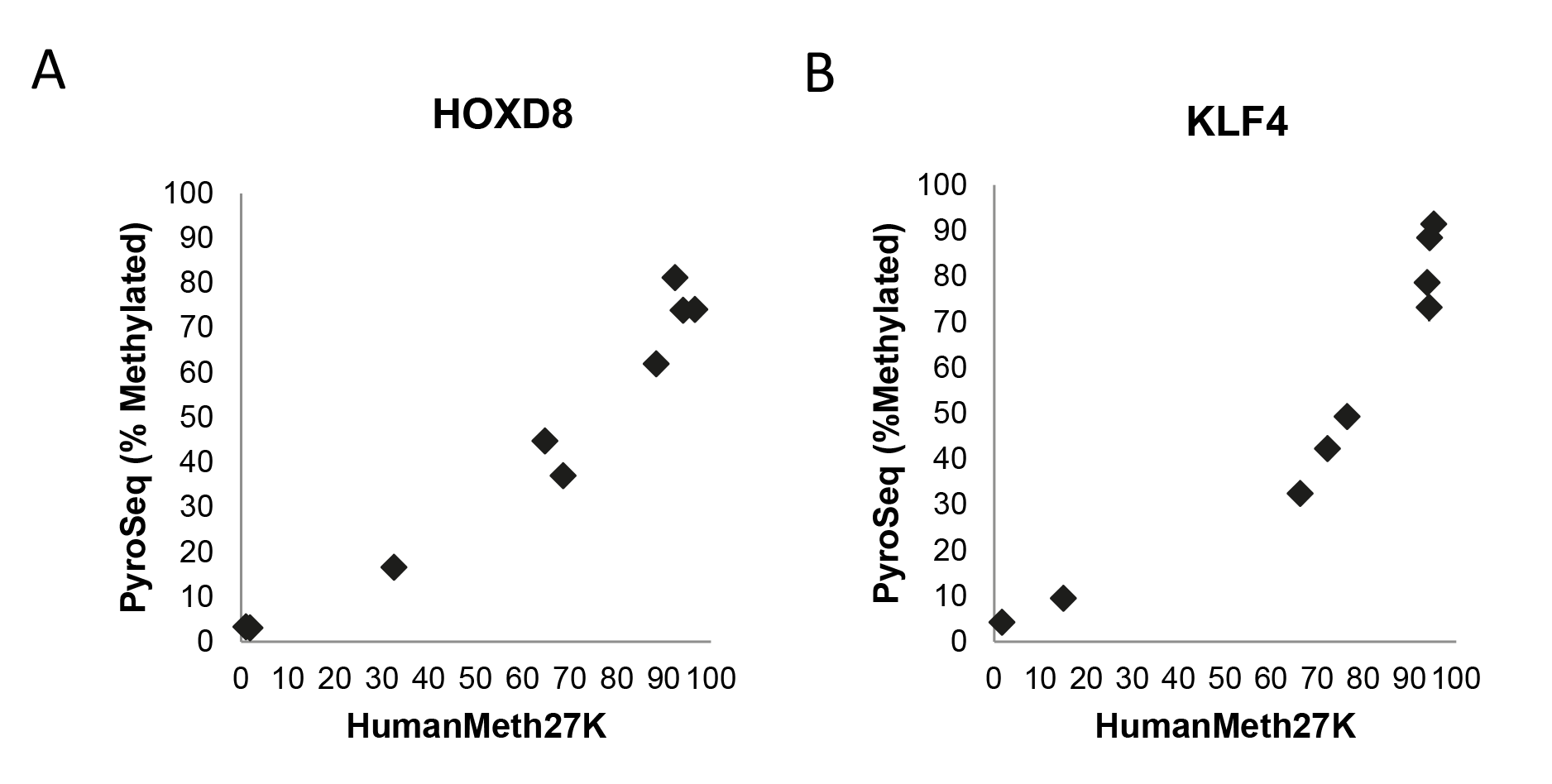

Supplement: Figure S6 — Verification of array data by pyrosequencing. The methylation status of selected gene promoters, HOXD8 (cg15520279), and KLF4 (cg07309102), was determined by pyrosequencing and compared with the HumMeth27K array data. (TIF) [file pone.0065373.s006.tif]

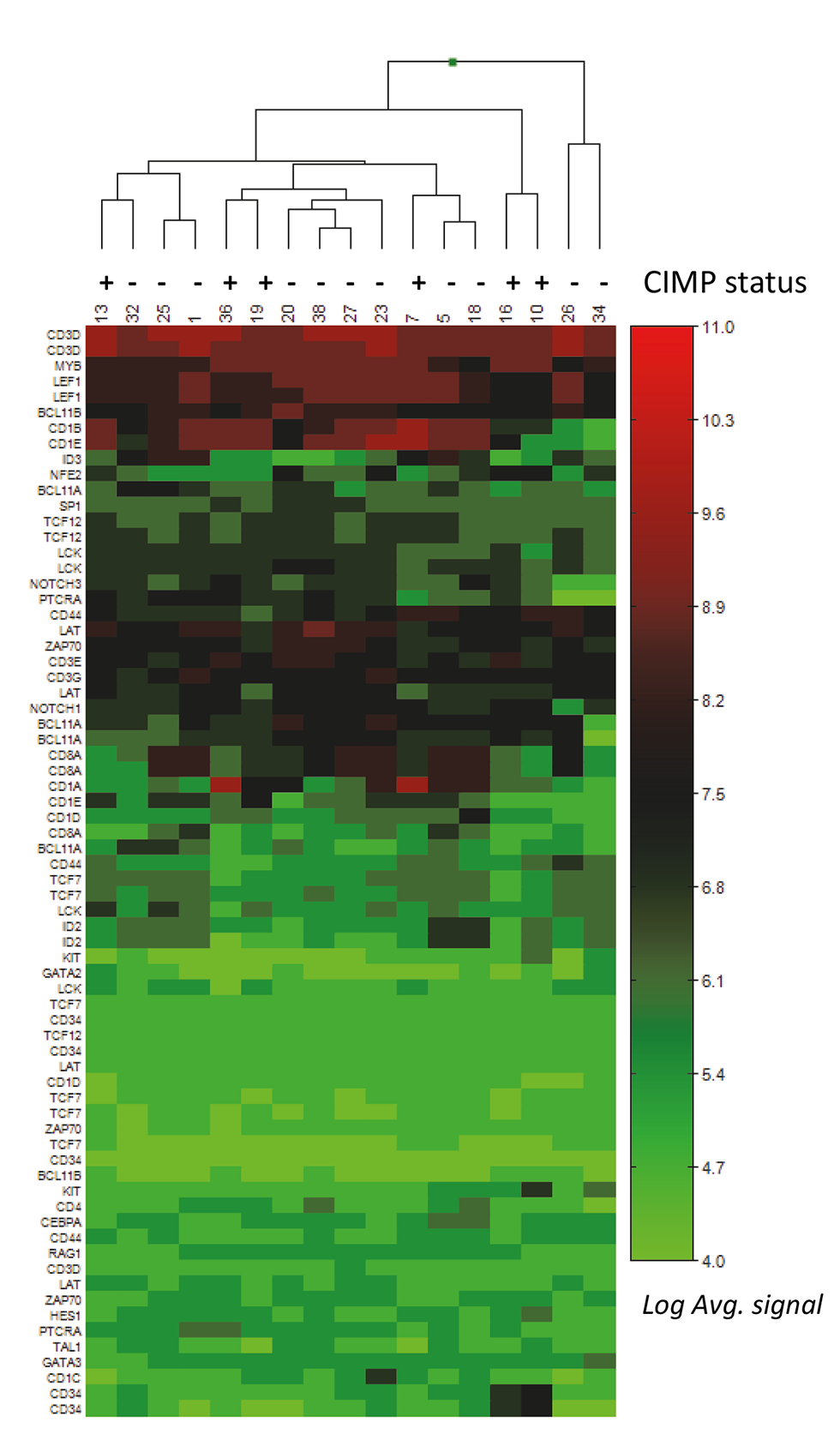

Supplement: Figure S7 — ETP gene signature analysis. The early T cell precursor (ETP) gene expression signature identified by Gutierrez et al. in 2010 [28] was used for analysis of 17 T-ALL samples analyzed by the HT-12 gene expression array. The heat map shows log. Average signal of the ETP associated genes for each sample. Euclidean hierarchical cluster analysis did not discriminate CIMP+ from CIMP− samples. (TIF) [file pone.0065373.s007.tif]
